# Factors correlating with delayed trauma center admission following traumatic brain injury

**DOI:** 10.1186/1757-7241-21-67

**Published:** 2013-09-10

**Authors:** Rahul Raj, Jari Siironen, Riku Kivisaari, Markku Kuisma, Tuomas Brinck, Jaakko Lappalainen, Markus B Skrifvars

**Affiliations:** 1 Department of Neurosurgery, Helsinki University Central Hospital, Helsinki, Finland; 2 Department of Anesthesiology, Intensive Care, Emergency Care and Pain management, Helsinki University Central Hospital, Helsinki, Finland; 3 Department of Orthopedics and Traumatology, Helsinki University Central Hospital, Helsinki, Finland

**Keywords:** Traumatic brain injury, Pre-hospital, Transport, Triage, Outcome, Emergency medical service

## Abstract

**Background:**

Delayed admission to appropriate care has been shown increase mortality following traumatic brain injury (TBI). We investigated factors associated with delayed admission to a hospital with neurosurgical expertise in a cohort of TBI patients in the intensive care unit (ICU).

**Methods:**

A retrospective analysis of all TBI patients treated in the ICUs of Helsinki University Central Hospital was carried out from 1.1.2009 to 31.12.2010. Patients were categorized into two groups: direct admission and delayed admission. Patients in the delayed admission group were initially transported to a local hospital without neurosurgical expertise before inter-transfer to the designated hospital. Multivariate logistic regression was utilized to identify pre-hospital factors associated with delayed admission.

**Results:**

Of 431 included patients 65% of patients were in the direct admission groups and 35% in the delayed admission groups (median time to admission 1:07h, IQR 0:52–1:28 vs. 4:06h, IQR 2:53–5:43, p <0.001). In multivariate analysis factors increasing the likelihood of delayed admission were (OR, 95% CI): male gender (3.82, 1.60-9.13), incident at public place compared to home (0.26, 0.11-0.61), high energy trauma (0.05, 0.01-0.28), pre-hospital physician consultation (0.15, 0.06-0.39) or presence (0.08, 0.03-0.22), hypotension (0.09, 0.01-0.93), major extra cranial injury (0.17, 0.05-0.55), abnormal pupillary light reflex (0.26, 0.09-0.73) and severe alcohol intoxication (12.44, 2.14-72.38). A significant larger proportion of patients in the delayed admission group required acute craniotomy for mass lesion when admitted to the neurosurgical hospital (57%, 21%, p< 0.001). No significant difference in 6-month mortality was noted between the groups (p= 0.814).

**Conclusion:**

Delayed trauma center admission following TBI is common. Factors increasing likelihood of this were: male gender, incident at public place compared to home, low energy trauma, absence of pre-hospital physician involvement, stable blood pressure, no major extra cranial injuries, normal pupillary light reflex and severe alcohol intoxication. Focused educational efforts and access to physician consultation may help expedite access to appropriate care in TBI patients.

## Introduction

Traumatic brain injury (TBI) is the leading cause of death and disability among the young around the world [1]. The American College of Surgeons Committee on Trauma and Centers for Disease and Prevention have developed field triage guidelines for TBI patients, which are in conjunction with the Brain Trauma Foundation’s pre-hospital guidelines, internationally acknowledged cornerstones of pre-hospital TBI triaging and treatment [2,3]. The guidelines recommend direct transport of patients with TBI to hospitals with availability of neurosurgical care including computerized tomography (CT) scanning, neurosurgical care, intracranial monitoring and treatment [3]. Accordance with the guidelines has been shown to improve outcome in TBI patients [4-6]. Despite this nearly half of all TBI patients are initially transported to a hospital without neurosurgical expertise, before inter-transfer to a hospital with neurosurgical capability, which has been shown to increase mortality [5,7].

Accordingly, we sought to assess pre-hospital factors associated with an initial transport to a non-neurosurgical hospital in a region where all TBI care is centralized to one single trauma center.

## Methods

### Hospital and trauma system

Finland is divided into five major health care regions each having a three-tier health care system: local district hospitals (primary care), regional central hospitals (secondary care) and university hospitals (tertiary care). The Helsinki health care region is the largest health care region in the country serving a population of nearly two million people. There are over twenty different primary and secondary level of care hospitals in the region but only one tertiary level of care hospital providing neurosurgical care (Töölö Hospital, Helsinki University Central Hospital, referred to as “TC” in the text). Accordingly, all TBI care is centralized to the designated TC. Pre-hospital emergency medical service (EMS) protocol mandates transportation of patients requiring neurosurgical and intensive care directly to the designated hospital following injury. The EMS routinely stabilizes the patients in the field (such as controlling of the airway in unconscious patients, correction of hypotension and hypoxia) enabling direct transport throughout the region when indicated. The EMS may always consult a pre-hospital physician and request for assistance and/or transport instructions.

### Patients and data collection

A retrospective analysis of all TBI patients (adults and children) admitted to the Töölö Hospital (Helsinki University Central Hospital) trauma intensive care units (ICU) during a two-year period (1.1.2009 - 31.12.2010) was conducted. Patients were categorized into two groups: direct admission and delayed admission. Patients in the delayed admission group were initially transported to a local hospital without neurosurgical expertise before inter-transfer to the TC.

The hospital and EMS records were reviewed for data regarding reason for dispatch urgency, injury details, patient characteristics, time details, transport destination and pre-hospital physician involvement. Time of injury was defined as time of dispatch call unless other mentioned in the EMS records. Dispatch urgency is ranging from A to D (A being the most urgent and D the least urgent) and categorized as urgent (A-B) and non-urgent (C-D). Patient blood alcohol level (BAL) was measured in the pre-hospital setting using an alcohol breath test and categorized to: none (0.0‰), low (< 2.3‰), high ≥ 2.3‰ and not tested [8]. The blood alcohol breath test has been shown to be a reliable test for measuring blood alcohol concentration [9]. Glasgow coma scale (GCS) was measured in the field and upon trauma center admission and categorized as moderate-to-severe (3–12) and mild (13–15) [10]. High-energy injury was defined as velocities over 25 km/h or head hitting the ground from above two meters height. Hypoxia and hypotension are defined according to the BTF guidelines: oxygen saturation <90% or systolic blood pressure <90 mmHg at any time during the pre-hospital transport [3]. Major extra cranial injury was collected from EMS forms, and defined retrospectively as any extra cranial injury that requires hospital admission within its own rights [11]. Patient head CT was classified by a radiologist and neurosurgeon (RK) unaware of EMS care according to the Rotterdam CT-score [12]. Overall injury severity and TBI severity were retrospectively evaluated using the injury severity score (ISS) and the IMPACT-TBI model [13,14]. Patient outcome was 6-month mortality.

### Statistical analysis

For statistical analysis: “IBM Corp. Released 2011. IBM SPSS Statistics for Windows, Version 20.0. Armonk, NY: IBM Corp” was used. For univariate categorical analysis the *χ*^2^ test (two-tailed) was used. Continuous variables were analyzed for normality distribution. All continuous data were highly skewed; the non-parametric Mann–Whitney *U* test was used. Data is presented as median values with interquartile range (IQR), unless other is mentioned. Significant variables (p ≤ 0.05) from the univariate analysis were used to build a logistic regression model to identify variables present at scene of associated with pre-hospital transport location. Only factors available at the scene of the incident were included (with the exception of penetrating head injuries as all penetrating head injuries are automatically transported to the designated trauma center). Collinearity between variables was tested for by assessing the variance inflation factor (VIF). Missing values were categorized separately but included in the models.

## Results

### Baseline characteristics

A total 431 were included patients of whom 65% were in the direct admission group and 35% in the delayed admission group. Patient median age was 54 (32– 64) and 71% were male (Table 1)*.* Median time from injury to TC admission was 1:07h (0:52–1.28) for the directly admitted patients versus 4:06h (2:54–5:43) for the delayed patients (p<0.001). The two most common causes of injury were ground level fall accidents (GLF) and road traffic accidents (RTA) (49%, 21%). Ground level falls were more frequent in the delayed admission group (70%, 35%, p< 0.001) and RTA more frequent in the direct admission group (32%, 3%, p< 0.001). High-energy traumas weresignificantly more often noted among the directly admitted patients (45%, 2%, p< 0.001). Also, pre-hospital physician involvement was significantly more noted in the direct admission group (p< 0.001).

**Table 1:** Patients baseline characteristics and injury details

		**Trauma center admission**		
	**All patients 431 (100%)**	**Direct 282 (65%)**	**Delayed 149 (35%)**	**p-Value**
**Age**	54 (32–64)	49 (24–64)	59 (47–66)	< 0.001
**Male gender**	307 (71)	188 (67)	119 (80)	0.004
**Location of the incident**				
Home	107 (25)	61 (22)	46 (31)	< 0.001
Public place inside	55 (13)	37 (13)	18 (12)	
Public place outside	205 (48)	157 (56)	48 (32)	
Other / Unknown	64 (14)	27 (9)	37 (25)	
**Time of incident**				
Weekday	279 (65)	181 (64)	98 (66)	0.743
Weekend	152 (35)	101 (36)	51 (34)	
8-17 (office hours)	206 (48)	126 (45)	80 (54)	
17-8 (outside office hours)	225 (52)	156 (55)	69 (46)	
Injury energy*				
High	130 (30)	127 (45)	3 (2)	< 0.001
Low	301 (70)	155 (55)	146 (98)	
**Penetrating injury**	6 (1)	6 (2)	0 (0)	0.073
**Physician consulted**	86 (20)	59 (21)	27 (18)	< 0.001
**Physician present**	208 (48)	184 (65)	24 (16)	< 0.001
**EMS risk category**				
A-B	304 (71)	230 (82)	74 (50)	< 0.001
C-D	74 (17)	32 (11)	42 (28)	
Missing	53 (12)	20 (7)	33 (22)	
**Time intervals (hh:min)**				
Injury to EMS arrival	0:10 (0:07–0:16)	0:10 (0:07–0:17)	0:10 (0:07–0:16)	0.750
Injury to first hospital	1:05 (0:49–1:27)	1:07 (0:52–1:28)	1:03 (0:43–1:24)	0.177
Time spent at other hospital			3:03 (1:51–4:50)	
Injury to trauma center	1:23 (0:58–2:51)	1:07 (0:52–1:28)	4:05 (2:53–5:43)	< 0.001

Categorical variables are presented as N (%) and continuous variables as median (IQR).

Abbreviations: TBI= Traumatic Brain Injury, EMS= Emergency Service Personnel.

*High-energy traumas are defined as velocities over 25 km/h or falls from over two meters height.

### Pre trauma center clinical features

Patients in the direct admission group had a significantly lower field GCS than patients in the delayed admission group (p< 0.001) (Table 2). However, there were no differences in GCS when admitted to the TC (p= 0.063) (Table 3). In univariate analysis direct admission showed significant association with: documented unconsciousness (p< 0.001), major extra cranial injury (p< 0.001), hypoxia (p< 0.001), hypotension (p< 0.001) and abnormal pupillary light reflex (p< 0.001).

**Table 2:** Differences in initial pre-hospital clinical features made by the emergency medical service between patients transported directly and indirectly to the trauma center

		**Trauma center admission**		
	**All patients 431(100%)**	**Direct 282 (65%)**	**Delayed 149 (35%)**	**p-Value**
**Field GCS**				
3-12	258 (60)	204 (72)	54 (36)	< 0.001
13-15	155 (36)	74 (27)	80 (54)	
Missing	18 (4)	3 (1)	15 (10)	
**Major extra cranial injury**	118 (27)	107 (38)	11 (7)	< 0.001
**Unconsciousness**	260 (60)	198 (70)	62 (42)	< 0.001
**Pupillary light reaction**				
Normal	284 (66)	181 (64)	103 (69)	< 0.001
Abnormal	87 (20)	76 (27)	11 (7)	
Missing	60 (14)	25 (9)	35 (24)	
**Hypoxia**	87 (20)	72 (26)	15 (10)	< 0.001
**Hypotension**	49 (11)	47 (17)	2 (1)	< 0.001
**Field blood alcohol level** (‰)				
0.0	17 (4)	13 (5)	4 (3)	0.004
< 2.3	44 (10)	28 (10)	16 (11)	
≥ 2.3	38 (9)	15 (5)	23 (15)	
Not tested	332 (77)	226 (80)	106 (70)	
Median	2.0 (0.9-2.8)	1.7 (0.6-2.4)	2.3 (1.6-2.9)	0.008
**Glucose** (mmol/l)	7.2 (6.0-8.8)	7.2 (6.0-8.9)	7.1 (5.9-8.8)	0.773
**Focal neurological sign**	37 (9)	24 (9)	13 (9)	0.904

Categorical variables are presented as N (%) and continuous as median (IQR).

Abbreviations: GCS= Glasgow Coma Scale, EMS= Emergency Medical Service, EMS risk category A= highest risk and D= lowest risk. * Any injury that requires hospital admission within its own right.

† Hypoxic insult is defined as documented 0_2_ saturation < 90% at any time during the pre-hospital transfer.

‡ Hypotensive insult is defined as systole < 90 mmHg at any time during the pre-hospital transfer.

**Table 3:** Differences in injury severity after trauma center admission

		**Trauma center admission**		
	**All patients 431 (100%)**	**Direct 282 (65%)**	**Delayed 149 (35%)**	**p-Value**
**IMPACT-TBI score**	26 (14–47)	26 (12–50)	25 (16–41)	0.619
**Trauma center GCS**				
3-12	295 (68)	203 (72)	92 (62)	0.063
13-15	136 (31)	79 (28)	57 (38)	
**Rotterdam CT-score**				
1-2	93 (21)	59 (21)	34 (23)	0.584
3-4	251 (59)	163 (57)	88 (59)	
5-6	87 (20)	60 (22)	27 (18)	
**ISS > 15**	372 (86)	235 (83)	137 (92)	0.013
**ISS > 25**	160 (37)	111 (39)	49 (33)	0.186
**Acute craniotomy**	144 (33)	59 (21)	85 (57)	< 0.001

Categorical variables are presented as N (%) and continuous variables as median (IQR). *Abbreviations*: CT= computer tomography, ISS= injury severity score, IMPACT= international mission for prognosis and clinical trials on TBI.

Furthermore, patient BAL was significantly associated with indirect transport in univariate analysis as 61% of patients with a high BAL were indirectly transported to the trauma center, compared to 36% and 24% for patients with low and negative BAL (p= 0.017). Median time from injury to TC admission for patients with a high BAL was 2:53h (1:17–7.27), which was significantly longer than for patients with a low BAL (1:20h, 0:58–2:55), negative BAL (1:23h, 1:07–1:43) and not tested (1:19h, 0:56–2:25) (p= 0.006). As only 23% of patients were tested for BAL in the pre-hospital setting we tested the correlation between the pre-hospital BAL and the blood alcohol level measured in the trauma center. Following trauma center admission, 48% of all patients were tested for blood alcohol levels. The BAL measured in the pre-hospital setting correlated extremely well with the blood alcohol levels measured in the trauma center (Spearman’s correlation coefficient: 0.844, p< 0.001).

Patients in the direct admission group were significantly more often intubated in the field compared to patients in the delayed admission group (N= 158/282, 56%; N= 7/149, 5%; p< 0.001); a pre-hospital physician was present in 84% (N= 133/158) of cases in the direct admission group and in 43% (N= 3/7) of cases in the delayed admission group. Additionally 58 patients in the delayed admission group were intubated at the local hospital before transfer to the TC. Thus, on TC admission 44% (N= 65/149) of patients in the delayed admission group were intubated compared to 56% (N= 160/282) of patients in the direct admission group (p= 0.010). Furthermore, of the 149 patients in the delayed admission group 6% (N= 8) had a documented episode of hypoxia (O_2_< 90%) and 1% (N= 2) a documented episode of hypotension (systolic blood pressure< 90 mmHg) at the local hospital before TC transfer. Mean time to TC admission for patients initially transported to a local hospital where they were intubated before transfer to the TC was 4:30h (IQR 3:19–6:08), which did not significantly differ from those not intubated at the local hospital before transfer to the TC (median 4:02h, IQR 2:50–5:38) (p= 0.389).

### Pre-hospital transport

In a multivariate logistic regression analysis factors increasing likelihood of delayed admission were: male gender (OR: 3.82, 95% CI: 1.60-9.13, p= 0.003), incident at public place compared to home (OR: 0.26, 95% CI: 0.11-0.61, p= 0.002), high energy trauma (OR: 0.05, 95% CI: 0.01-0.28, p= 0.001), pre-hospital physician consultation (OR: 0.15, 95% CI: 0.06-0.39, p< 0.001) or presence (OR: 0.08, 95% CI: 0.03-0.22, p< 0.001), hypotension (OR: 0.09, 95% CI: 0.01-0.93, p= 0.044), major extra cranial injury (OR: 0.17, 95% CI: 0.05-0.55, p= 0.003), abnormal pupillary light reflex (OR: 0.26, 95% CI: 0.09-0.73, p= 0.010) and severe alcohol intoxication (OR: 12.44, 95% CI: 2.14-72.38, p= 0.005) (Table 4). No collinearity in the final model was noted (VIF_max_= 2.1).

**Table 4:** Multivariate analysis showing pre-hospital factors associated with direct trauma center admission

**Variable**	**OR (95% CI)**	**p-Value**
**Age**	1.02 (1.00-1.04)	0.098
**Male**	3.82 (1.60-9.13)	0.003
**EMS dispatch urgency**		
Urgent (A-B)	1.0	
Non-urgent (C-D)	0.75 (0.33-1.71)	0.500
**Incident location**		
Home	1.0	
Public place	0.26 (0.11-0.61)	0.002
Other/Unknown	2.15 (0.70-6.59)	0.181
**Injury energy**		
Low	1.0	
High	0.05 (0.01-0.28)	0.001
**Pre-hospital physician**		
Consulted	0.15 (0.06-0.39)	< 0.001
Present (called to the scene)	0.08 (0.03-0.22)	< 0.001
**Documented unconsciousness**	0.62 (0.27-1.41)	0.256
**Hypoxia**	1.02 (0.42-2.44)	0.972
**Hypotension**	0.09 (0.01-0.93)	0.044
**Major extra cranial injury**	0.17 (0.05-0.55)	0.003
**Abnormal pupillary light reflex**	0.26 (0.09-0.73)	0.010
**Field GCS**		
3-12	1.0	
13-15	1.64 (0.72-3.76)	0.240
**Blood alcohol level (‰)**		
0.0	1.0	
< 2.3	0.99 (0.19-5.33)	0.995
> 2.3	12.44 (2.14-72.38)	0.005

Odds ratios over one indicating an increased likelihood of direct admission and odds ratios under one indicating a decreased likelihood of direct admission (i.e. increased likelihood of delayed admission). *Abbreviations*: OR= odds ratio, CI= confidence interval, GCS= glasgow coma scale, EMS= emergency medical service.

### Post trauma center admission

Following TC emergency department admission and resuscitation 82% patients in the direct admission group were intubated compared to 81% in the delayed admission group (p= 0.870). There was no significant difference in admission Rotterdam CT-score between the groups (p= 0.584) (Table 3). Although, subdural hematoma and midline shift was more frequent in the delayed admission groups (p< 0.001) and traumatic subarachnoid hemorrhage more frequent in the direct admission group (p= 0.003). A significant higher proportion of patients in the delayed admission group underwent acute craniotomy when admitted to the trauma center (57%, 21%, p < 0.001). Also, an ISS > 15 were more frequently among patients in the delayed admission group compared to the direct admission group (92%, 83%, p= 0.013). However, no differences ISS > 25 were noted between the groups (p= 0.186). According to the IMPACT-TBI model, no significant difference in median TBI severity score was noted between the direct admission group (24, IQR: 12–50) and delayed admission group (25, IQR: 16–41) (p= 0.657). Univariate analysis showed no significant difference in 6-month mortality between the groups (24%, 25%, p= 0.814). There was no significant difference in time from injury to TC admission between survivors and non-survivors (p= 0.331).

## Discussion

The centralization of TBI care to specialized trauma centers have improved outcome [15,16]. Direct transport of patients, from the scene of injury, to such specialized trauma centers has been shown to significantly increase survival [5,6,15]. Thus, initial pre-hospital triage and subsequent transport is of major role in the handling of TBI patients. The purpose of this study was to investigate pre-hospital factors associated with an inappropriate transport of TBI patients in a region where all TBI care is centralized to one single trauma center. Our study showed that despite a regional trauma care protocol, mandating direct transport of TBI patients that are likely to specialized care to the TC, over one third are initially transported to a hospital without the capacity of handling these patients. We identified several factors associated with transport to another hospital than the designated trauma center, i.e. male gender, low energy of trauma, lack of pre-hospital physician involvement, absence of major extra cranial injuries, normal pupillary light reflex and severe alcohol intoxication. Our re-transfer rate is somewhat lower than previously described, indicating a well-organized system in the region [7,17]. A probable major factor influencing this is the availability of pre-hospital physicians in the study region.

Several studies have showed an improved outcome after direct admission to a neurosurgical trauma center, although there is no class I evidence to back this up. Härtl et al. showed that patients transported indirectly to a neurosurgical trauma center, via a lower level of care hospital, had a 50% higher risk of death than patients transported directly to a trauma center with neurosurgical expertise [5]. Hannan et al. reported an almost twice as low risk for in patients with TBI treated in a regional trauma center compared to local non-trauma centers [19]. In our study, we did not find any association between initial transport destination and outcome. Despite the fact that patients who were initially transported to a local hospital (delayed admission) were more severely injured than the patients in the direct admission groups. Further sub-group analysis showed that patients in the delayed admission group more often required acute craniotomy than patients in the direct admission group. As such one might expect a higher mortality among these patients, as it has been shown in numerous studies before [20-23]. However, this as well was not evident in our study. The reason why we could not find any correlation between initial transport destination and outcome is probable due to lack of power. Another possible reason might be that even though the indirectly transported patients had a significantly longer time delay to trauma center admission than the directly transported patients the delay wasn’t long enough to affect outcome. This is supported by the fact that the majority of patients in both the direct and delayed admission group undergoing acute craniotomy was operated on well within acknowledged time limits [18,24]. Moreover, we found no significant difference in time to trauma center admission between survivors and non-survivors. This supports the earlier statement regarding a well working trauma system in the region. Another factor and major strength to the present study that has to be considered is that we used 6-month mortality as outcome measure whereas most other studies have used in-hospital mortality. In-hospital mortality is not an optimal marker for measuring TBI as it severely underestimates the number of deaths [11]. Thus, it may simply be that indirect transport increases the risk of short-term but not long-term mortality. However, no statistical significant difference in 14-day mortality was noted between the direct and delayed admission groups in univariate analysis.

Not surprisingly a high field GCS and the absence of major extra cranial injuries associated with delayed trauma center admission. However, field assessment of injury severity was proven not to be a reliable marker of injury severity as post-admission investigations revealed that patients in the delayed admission group had higher injury severity scores and that there were no differences in TBI severity, despite contrary signs in the pre-hospital setting. The GCS has been shown to be of limited value in determining TBI severity in the field [25]. Fourteen percent of patients with an initial GCS of 14 develops an intracranial lesion [26]. Intracranial lesions in patients with an initial GCS of 15 are uncommon unless risk factors are present, such as high age, use of anti-coagulants, focal neurological symptoms and alcohol-intoxication [27,28]. Approximately 3-6% of all TBI patients who die have an field GCS of 13 to 15 [29,30]. This is of significance in pre-hospital care as lucid patients with a high initial GCS easily can be under triaged and the possibility of an intracranial lesion overlooked. This problem was noted in our study as 31% of all patients with a field GCS of 13 to 15 required acute craniotomy. Furthermore, 15% of all patients with a field GCS of 13 to 15 died (compared to 32% of those with a field GCS of 3 to 12). It should however be noted that field GCS was measured by a paramedic if a pre-hospital physician was not present (direct admission 35%, delayed admission 84%) but TC GCS was always measured by an emergency department physician (trauma surgeon, anesthesiologist or neurosurgeon). This potential confounding factor in determining accurately determining the GCS should be the focus of future studies.

Systemic hypotension and hypoxia in the pre-hospital setting are associated with poor outcome in TBI patients [3]. Twenty percent of all patients had a hypoxic insult (SO2< 90%) and eleven percent a hypotensive insult (< 90 mmHg) during the pre-hospital transport. In univariate analysis both hypotension and hypoxia were significantly associated with direct admission. However, in multivariate analysis hypotension but not hypoxia was showed significant association with direct admission. To further investigate this we looked at the number of hypotensive and hypoxic patients accompanied by a pre-hospital physician, as the presence of a physician was the strongest independent predictor of direct admission in the logistic regression analysis. However, we found no significant difference in hypotensive respectively hypoxic patients accompanied by a physician (76%, 70%).

Securing the airway and adequate oxygenation and ventilation is of vital role in the pre-hospital management of patients with TBI [3]. A significant higher proportion of patients in the direct admission group were intubated on the field compared to patients in the delayed admission group. Of all patients intubated in the field 82% were accompanied by a physician (3 out of 7 in the delayed admission group and 133 out of 158 in the direct admission group). However, a considerable proportion of patients in the delayed admission group were intubated at the local hospital before transfer to the TC. Notable is that there were no significant difference in time to TC admission between patients intubated and not intubated at the local hospital before transfer to the TC.

We found that severe alcohol intoxication was associated with delayed admission. Alcohol intoxication in TBI patients is an internationally acknowledged problem [1]. Studies have shown that roughly half of TBI patients are alcohol-intoxicated at the time of injury and that alcohol intoxication increases the risk of TBI, especially as a result of binge drinking [31-34]. Alcohol has also been shown to impede with the clinical TBI diagnosis by having a level of consciousness lowering effect [25]. This makes it challenging for the EMS to judge whether a trauma patient’s altered level of consciousness (i.e. GCS) is caused by alcohol intoxication, intracranial lesion or other injuries. Ground level falls is the major cause of injury among alcohol intoxicated patients and the elderly [35]. Our results show that low energy traumas, such as GLF, significantly increase the risk of delayed admission. Thus, GLF might be an underestimated cause of injury in TBI patients. Though, it has been shown that there is no difference in TBI severity caused by GLFs compared to other causes of injury, such as RTA [36]. Hence, the elderly and alcohol-intoxicated patients may be at the core of this problem; pre-hospital mistriage leading to delayed life-saving treatment.

Altogether, there are many contributing factors contributing to difficult triage decisions with the risk of both under or over triage of these patients. Over triaging is not a viable option as this would lead to an excess trauma center burdening and under triage with the risks of having devastating effects on patient prognosis. It is in such cases that the importance of pre-hospital physician consultation is highlighted. We acknowledge the fact that not all regions have pre-hospital physicians available 24/7, however in areas of high trauma burdening this could be an opportunity worth exploring.

In conclusion our study shows that some patients with TBI are often misdiagnosed in the field due to a number of reasons and that there is a major need for further studies exploring diagnostic and educational means to improve rapid identification of those requiring neurosurgical treatment in patients whose initial clinical presentation is confounded by factors such as low level of initial symptoms and alcohol intoxication.

### Study limitations

Our study has several limitations. Most important, due to the retrospective nature of this study we were unable to collect the number of patients with a TBI requiring intensive care initially transported to another hospital were the patient died before reaching the trauma center’s emergency department. Likewise we are unaware of the number patients dying at the scene of injury before reaching the TC, or at the local hospital prior to trauma center transfer. However, in a large prospective study by Myburgh et al. they found that only 3% of patients with TBI die during the pre-intensive period [7]. We believe that these numbers are representative for our study as it is very rare that trauma patients requiring neurosurgical expertise are not referred to the designated TC (directly or indirectly) since the other hospitals do not offer any neurosurgical services. Second, BAL is not routinely tested for in the pre-hospital setting without indications, giving us a substantial amount of patients untested for BAL. It is clear that BAL testing is not indicated nor is it possible for everyone and this is probably the current situation in most countries where BAL is mainly tested when it might impact treatment decisions. Furthermore, measuring BAL in the pre-hospital setting using an alcohol breath-test is challenging in TBI patients considering the nature of the disease (potential lack of co-operation and unconscious patients). However, following TC admission a total of 48% of patients were tested for blood alcohol levels using standard laboratory blood samples. This was shown to correlate extremely well with the BAL measured in the pre-hospital setting. Third, in the present retrospective study we could not note any statistical significant association between initial transport location and long-term mortality, which is probably a consequence of lack of power.

## Conclusion

Delayed trauma center admission following TBI is common. Factors increasing likelihood of this were: male gender, incident at public place compared to home, low energy trauma, absence of pre-hospital physician involvement, stable blood pressure, no major extra cranial injuries, normal pupillary light reflex and severe alcohol intoxication. These data suggest that educational efforts for the EMS about the common clinical reasons impeding with early TBI diagnosis may help expedite access to appropriate level of care for such patients are warranted. These data also highlight the need for improved diagnostic tools for identifying cases that require immediate neurosurgical intervention in patients whose clinical picture is confounded by minimal initial symptoms and especially alcohol intoxication.

## Competing interests

The authors declare that they have no competing interests.

## Authors’ contribution

RR, JS and MS designed the study. RR drafted the manuscript assisted by MS, JS and JL, RR, TB and RK performed the data collection. RR is responsible for integrity of the collected data. The statistical analysis of the data was performed and interpreted by RR, JS and MS. RK viewed and interpreted CT scans of the included patients. MS, JL contributed to the interpretation of the data and writing of the manuscript. All authors revised the manuscript and approved it in the final form.
